# Nurses’ Evaluation of a Service Robot for Inpatient Care: Technology Acceptance Study

**DOI:** 10.2196/86824

**Published:** 2026-04-14

**Authors:** Christopher Friese, Robert Klebbe, Anika Heimann-Steinert

**Affiliations:** 1 Department of Geriatrics and Medical Gerontology, Charité – Universitätsmedizin Berlin, corporate member of Freie Universität Berlin and Humboldt-Universität zu Berlin, Augustenburger Platz 1, Berlin, 13353, Germany, 49 30450553796; 2 Institute of Public Health, Charité – Universitätsmedizin Berlin, corporate member of Freie Universität Berlin and Humboldt-Universität zu Berlin, Berlin, Germany

**Keywords:** nursing, inpatient care, digital health, assistive technology, robotics, service robotics, technology acceptance, human-robot interaction, user-centered design

## Abstract

**Background:**

The integration of robotic systems into nursing practice is increasingly discussed as a potential strategy to alleviate workload and support care processes in response to demographic changes and staffing shortages. However, the acceptance of nursing staff as primary end users remains a critical determinant for successful implementation. Despite technological advances, the practical requirements and perspectives of nursing staff have not been adequately considered in research and development efforts to date.

**Objective:**

Building on the user-centered development approach applied, this study aimed to examine nursing staff’s evaluation of a service robot designed to assist with routine tasks in inpatient care, as well as their intention to use it, while accounting for technology-specific and psychological determinants of acceptance.

**Methods:**

A total of 30 nurses tested the robot across 3 application scenarios (information service, item delivery, and beverage delivery) in a simulated care setting, alternating between the roles of nurse and care recipient. Acceptance-related constructs, including intention to use, were measured using the Technology Usage Inventory. General attitudes toward robots were assessed via the General Attitudes Towards Robots Scale. Participants’ prior experience with robotics was also documented. Spearman rank correlations and Mann-Whitney *U* tests were used for analysis.

**Results:**

The robot was rated positively across all dimensions. Usability was high (median 20, IQR 18-21; scale range 3‐21), as was perceived usefulness (median 21, IQR 16-24; range 4‐28). Skepticism was low (median 10.5, IQR 7-12; scale range 4‐28), and accessibility was moderate (median 10, IQR 8-13; scale range: 3‐21). Intention to use was strong (median 224.5, IQR 157-248; scale range 0‐300) and correlated positively with usability (*r_s_*(28)=0.505; *P*=.004), perceived usefulness (*r_s_*(28)=0.74; *P*<.001), and accessibility (*r_s_*(28)=0.628; *P*<.001), and negatively with skepticism (*r_s_*(28)=–0.516; *P*=.004). More positive personal attitudes toward robots were also associated with higher perceived usefulness (*r_s_*(28)=0.549; *P*=.002) and greater intention to use (*r_s_*(28)=0.483; *P*=.007). No significant differences in intention to use were found between participants with and without prior robotics experience (*U*=83.5; *P*=.62).

**Conclusions:**

The findings indicate a high level of acceptance among the participating nursing staff for the developed service robot within the tested scenarios. Considering the chosen user-centered development approach, they further underscore the need for strategies that combine participatory design, transparent communication of system capabilities and limitations, and structured opportunities for hands-on experience. Such measures, together with proactive knowledge transfer and skills development, are essential to sustainably leverage the practical potential of service robotics in nursing practice.

## Introduction

### Background

Against the backdrop of an aging population and a shortage of nursing staff [1,2], robotic assistance is being discussed as a promising form of support in inpatient and hospital care [3,4]. A recent scoping review by Ohneberg et al [5] identified assistive robotic systems in nursing and categorized applications into domains, including information and data-related support, monitoring and safety functions, communication and telepresence, and task-oriented assistance in everyday care processes, alongside socially assistive functions targeting emotional care and cognitive support. Against this broader landscape, this paper focuses on service robotics as one class of assistive robotic systems in inpatient care.

### Service Robotics in Nursing: Scope and Status Quo

Service robotics denotes technical systems that support people in the partially or fully automated execution of services and tasks. In addition to information-processing and sensory functions, service robots can move autonomously or carry out complex tasks comprising several work steps and materials [5]. Current developments in the nursing context aim at deploying service robots to support administrative tasks such as digital patient data collection and assistance during medical rounds [6,7]; logistical functions, including the transport of medication, supplies, and laundry [8-10]; as well as physically demanding care activities like patient lifting, transferring, and hygiene-related procedures involving both personal care and environmental cleaning tasks [11-14]. At present, the use of assistive robotic systems in nursing remains heterogeneous and occurs primarily during development phases and pilot implementations, although wider practical implementation is anticipated in the coming years [5,15,16].

Early evidence suggests that diverse stakeholder groups, such as nursing staff, are generally open to the integration of robotic assistance, provided that the technology complements rather than replaces human care [4,17]. The expectation is for robots to act as collaborative assistants, reducing the high workload without fully substituting nurses’ tasks [18,19]. Their introduction, however, entails a fundamental transformation of nursing work processes [3,5,20]. Consequently, in light of these systemic changes, end user acceptance emerges as a critical factor in the development and deployment of assistive robotic systems in nursing, as it has been consistently identified as a key enabler for successful implementation, as well as for their effective and efficient long-term use [21,22].

### Conceptual Background: Technology Acceptance and Intention to Use

In technology research, acceptance is commonly defined as users’ willingness to use a technology for the tasks it is intended to support [23]. This broad definition is typically operationalized in technology-acceptance frameworks through users’ perceptions of a technology’s characteristics and its suitability for intended tasks and conditions of use. In turn, these perceptions shape intention to use, with intention to use serving as the most proximal predictor of actual system use [24,25].

A prominent intention-based framework is the technology acceptance model (TAM) by Davis [26]. The TAM explains intention to use primarily through perceived usefulness and perceived ease of use, linking these perceptions to attitudes toward using the technology and, ultimately, to behavioral intention. While the TAM has been widely applied in health care contexts, its comparatively narrow focus has been criticized for failing to capture the full complexity of technology acceptance in such settings [27]. This critique aligns with a growing body of research highlighting limitations of established acceptance models and calling for broader conceptual frameworks that account for both technical attributes and individual-level factors, a perspective supported by recent reviews [28,29] and empirical studies [30-32].

In contrast to TAM’s parsimonious focus, broader implementation-oriented models such as the unified theory of acceptance and use of technology extend the explanatory scope by incorporating social and organizational determinants relevant to real-world implementation [25]. Diffusion-of-innovation perspectives, in turn, address adoption dynamics over time at the system level [33]. These approaches, as well as more general behavioral and health-behavior frameworks such as the theory of reasoned action, the theory of planned behavior, or the health belief model, were not adopted because this study targets technology-specific acceptance perceptions in a workplace context rather than implementation processes or health behavior change [34-36].

### Nursing Staff Perspectives on Robotic Assistance

From this perspective, nursing staff perceptions and acceptance criteria are particularly relevant given their role as the primary end users of assistive robotic systems. Although nursing staff play a central role in implementation and everyday use of such systems, their perspectives have long been underrepresented in technology acceptance research [3,37]. Recent studies have begun to address this gap, offering initial insights into nurses’ acceptance criteria. However, findings to date point to a complex and multifactorial picture.

Consistent with prior technology acceptance research, the robot’s perceived usefulness remains pivotal. Nurses are more likely to adopt a system that demonstrably reduces workload by assisting with physically demanding tasks such as lifting, transferring, or routine deliveries [3,38,39]. Ease of use is equally critical, as increased operational complexity or system failure can offset intended benefits [40]. Acceptance is further shaped by perceptions of reliability and safety. Only a system that performs dependably and poses no risk to care recipients is likely to be entrusted with responsibilities in patient care [41].

Beyond these technology-related considerations, individual-level factors such as attitudes, perceptions, and prior experiences play a critical role. A key factor is nurses’ general attitude toward technology and robots in particular. Nurses who are technologically inclined or have had positive experiences with new technologies tend to be more receptive to robotic assistance [21,32,42,43]. Multiple studies, including a large-scale survey of nursing staff, have repeatedly found that prior experience and knowledge specifically related to robotics positively influence acceptance. Nurses with first-hand experience and greater confidence in operating such systems consistently evaluate their usefulness and applicability more positively [31,38,44].

Taken together, this evidence suggests that acceptance is not a static reaction to technological features, but a dynamic process shaped by individual attitudes, prior experiences, and progressive familiarization. Moreover, empirical findings highlight the importance of early end user involvement in fostering acceptance. When nurses are engaged in planning, adaptation, and decision-making processes, they are more likely to perceive the robot as useful and aligned with their workflow, which in turn strengthens their intention to use the system in everyday nursing practice [45].

### Rationale and Objectives

Building on the limited routine deployment of assistive robotic systems in nursing and the multifactorial acceptance determinants outlined above, further evidence is needed from hands-on evaluations of concrete prototypes that are aligned with nursing workflows and professional requirements. Involving nursing staff in participatory development and providing opportunities for direct interaction are widely considered crucial to avoid technology-driven implementation and to ensure that robotic systems fit the practical realities, values, and needs of nursing practice [5,19,20,41].

The research and development project RoMi (Roboterunterstützung bei Routineaufgaben zur Stärkung des Miteinanders in Pflegeeinrichtungen), funded by the German Federal Ministry of Education and Research, aimed to develop a mobile service robot to support routine nonclinical tasks in inpatient care, such as information provision and logistical activities. Nursing staff were actively involved in key phases of the project, including the examination of contextual and ethical prerequisites for robot use [46], investigations of preferences regarding robot design and interaction characteristics [47-49], and the codevelopment of application scenarios, capability requirements, and evaluation criteria for context-sensitive human-robot collaboration [19].

Following this user-centered development approach, the resulting functional prototype was evaluated in an end user study with nursing staff. The study examined participants’ general attitudes toward robots, their evaluation of the developed service robot, and their intention to use it. Accordingly, we conceptualized acceptance in terms of intention to use and its multifactorial determinants, integrating both technology-related appraisals and individual-level factors. This paper aimed to advance a differentiated understanding of the usage potential of service robots in nursing, as well as the factors influencing their acceptance and prospective integration within inpatient care settings.

## Methods

### Procedure

The study consisted of an online presurvey and a subsequent testing and evaluation phase of the developed service robot. To recruit participants, various inpatient care facilities were informed about the study, enabling interested individuals to contact the study team directly. Once interest was expressed, potential participants received the study information and consent form, followed by the scheduling of a telephone consultation. During this call, the study’s objectives, design, and procedures were reiterated, and the inclusion criteria were assessed. These included (1) minimum age of 18 years; (2) current or past occupation as a nurse, nursing assistant, geriatric nurse, geriatric nursing assistant, pediatric nurse, occupational therapist, remedial therapist, or physical therapist in inpatient care or daycare; and (3) sufficient proficiency in the German language.

After providing informed consent, participants received a link to the online presurvey, which gathered sociodemographic data and included the General Attitudes Towards Robots Scale (GAToRS). Appointments were then arranged for the testing and evaluation phase, which took place at the research laboratory of the Geriatrics Research Group at Charité—Universitätsmedizin Berlin. Each session involved 2 participants who alternated between the roles of nurse and care recipient. Participants first received structured instruction and hands-on training covering safety procedures as well as the use of the robotic system and its smartphone app for task management and status monitoring.

**Figure 1.**
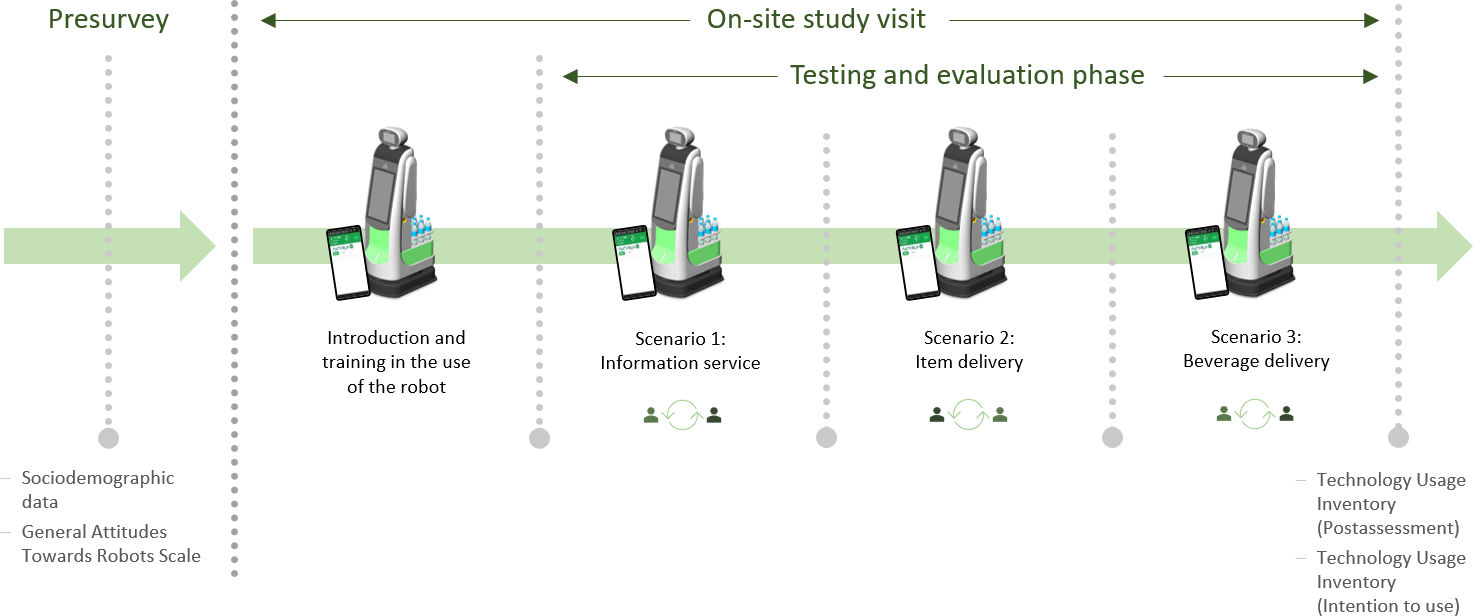
Study procedure.

The service robot was then tested and evaluated across three predefined application scenarios: information service, item delivery, and beverage delivery. These scenarios had been developed and refined in earlier empirical studies conducted within the RoMi project [19,46]. Each scenario was completed twice by every participant, once in each role. To ensure consistency, all received standardized written instructions outlining the required interaction procedures. After performing both roles within a scenario, the session proceeded to the next. Following the test phase, participants completed the posttest section of the Technology Usage Inventory (TUI), including the intention to use (ITU) scale, for the evaluation of the service robot. Each session lasted approximately 90 minutes. Figure 1 illustrates the overall study procedure.

### Robot

The service robot evaluated in this study was developed within the RoMi research project and represents an iteratively adapted prototype. A semihumanoid mobile service robot was selected to match the RoMi focus on routine, nonclinical support tasks, including information provision and logistics. Other systems such as patient-handling, telepresence, or socially assistive robots were not considered within RoMi because they address different task classes.

The developed service robot is based on the workerbot6 platform by pi4_robotics. Throughout the project, the platform was iteratively refined for the care setting, with technical implementation led by pi4_robotics. Iterative refinements were informed by empirical studies on robot design and interaction preferences led by Humboldt-Universität zu Berlin and by industrial design concepts contributed by Hochschule für Technik und Wirtschaft Berlin [47-49]. The authors affiliated with Charité—Universitätsmedizin Berlin contributed the user-centered workstream by conducting studies with nursing staff on practical and ethical considerations of robot use and translating the findings into the development and refinement of application scenarios, interaction sequences, and the associated capability requirements and evaluation criteria for the service robot [19,46].

**Figure 2.**
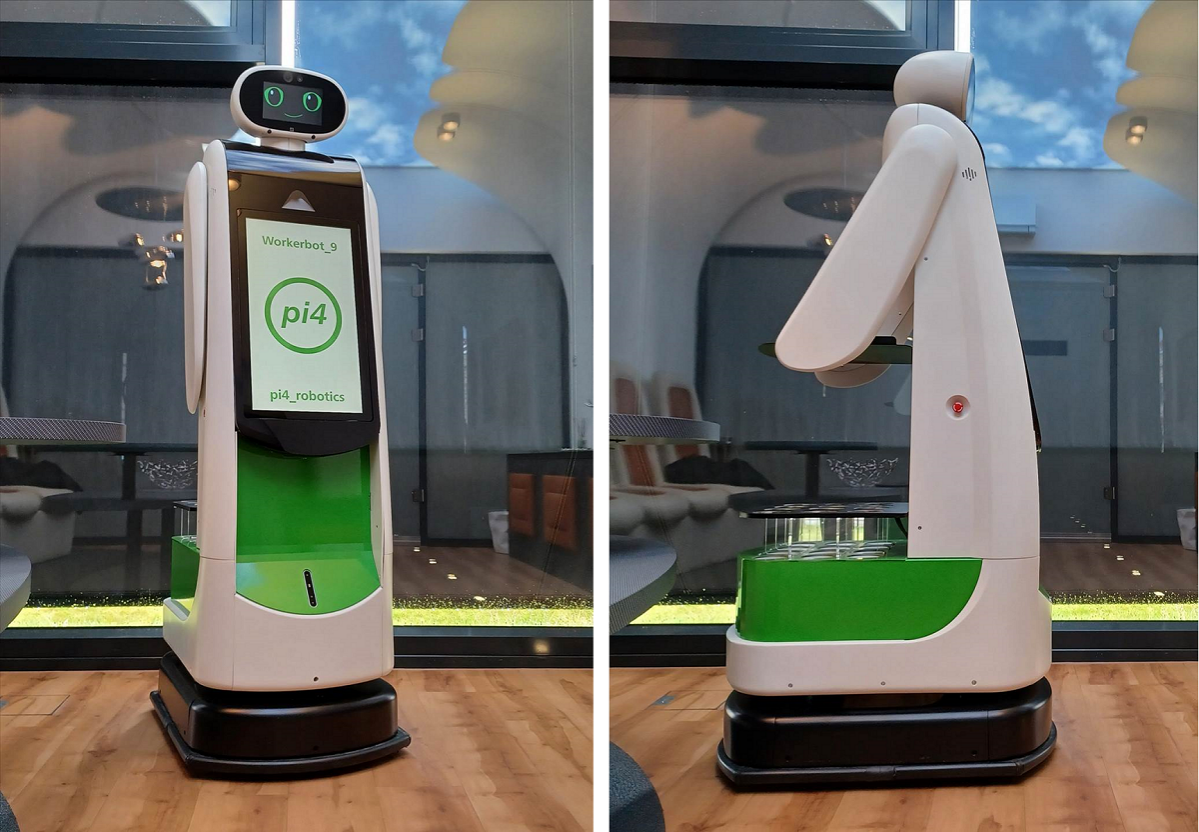
Developed service robot (image used with permission from pi4_robotics).

The research prototype ultimately resulting from the RoMi project and evaluated in this study is shown in Figure 2. The service robot has a height of 175 cm and a weight of 133 kg.

Based on initial design investigations showing that a human-like appearance can contribute to the acceptance and perceived competence of robots, an anthropomorphic embodiment was chosen [46,47]. The robot’s head unit features a screen displaying animated eyes and a mouth, as well as an integrated camera that detects human presence and enables context-sensitive autonomous interaction (eg, in patient rooms or at loading stations). A microphone for speech recognition is also embedded in the head section to enable low-effort voice input, which aligns with caregiver preferences for speech-based interaction [46-48].

The torso is equipped with a front-mounted 21.5-inch touchscreen display that provides graphical feedback on the robot’s status and serves as an additional interface for human-robot interaction. This visual channel was intended to enhance interaction transparency and comprehensibility while accommodating potential age- or disease-related changes in the communicative abilities of care recipients [46]. Loudspeakers and an emergency stop button are positioned on both sides of the robot to support clear feedback and immediate shutdown in case of safety concerns. Lateral, arm-like structures function as rails that support a fold-out tray mounted at the rear, enabling the transport and handover of small items without requiring complex manipulation. The back of the robot also houses a beverage dispenser, which is capable of holding twelve 0.5 liter bottles and is equipped with integrated sensors and compartment lighting to support structured loading and ensure correct retrieval during beverage delivery.

The robot is mounted on a mobile platform that enables autonomous navigation based on a map configurable for the ward environment. The robot’s navigation was configured with a safety-oriented low-speed profile (maximum 1.4 km/h) appropriate for shared indoor environments, consistent with RoMi project findings that caregivers perceive slower approach speeds as more comfortable and safer [49]. Moreover, the system incorporates a range of obstacle detection sensors. Upon encountering an obstacle, the robot first reduces speed and attempts to navigate around it. If avoidance is not possible, it stops and waits until the path is clear, thereby prioritizing safe behavior in the presence of uncertainty.

Throughout the study, participants used a smartphone app as the primary tool for giving instructions to the robot. The app also provided posttask status information.

### Setting

**Figure 3.**
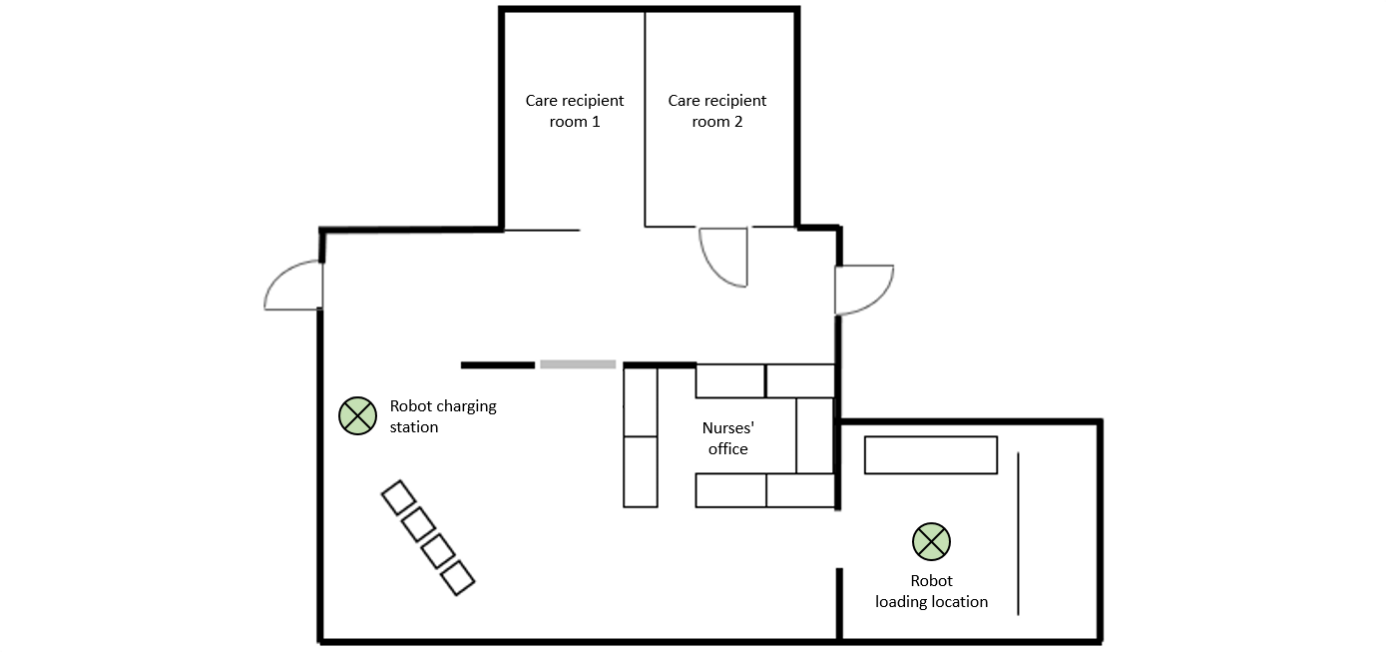
Setup within the laboratory setting.

To align the interaction with the robot more closely with the professional care context, a simulated nursing ward environment was set up in the research laboratory (Figure 3). The setup included a nurses’ office, 2 patient rooms, and a storeroom serving as the loading station. To minimize mutual influence, partition elements were installed to ensure that participants could not observe each other’s interactions while alternating roles.

### Application Scenarios

To standardize exposure to the robot, participants completed 3 scripted application scenarios that operationalized typical nonclinical support tasks and specified role-dependent interactions between the participant in the nurse role, the participant in the care recipient role, and the robot. Tasks were assigned via the robot’s instruction and status-monitoring app.

#### Information Service

The participant in the nurse role created a notification task in the app by selecting the room and entering a free-text message. The robot navigated to the room, signaled arrival with a bell sound, and waited until a person entered its detection area. After person detection, the participant in the care recipient role confirmed readiness to receive the notification via voice or touchscreen. The robot then delivered the message via audio and on-screen output and offered an option to repeat it. If no repetition was requested, the robot returned to the charging station.

#### Item Delivery

The participant in the nurse role initiated a delivery task in the app by selecting the room and entering a free-text item list. The robot navigated to a predefined loading area and, after person detection, provided voice and on-screen loading instructions. The participant in the nurse role placed the items on the rear tray and started execution on the touchscreen. The robot delivered to the room, signaled arrival, and prompted a confirmation-based handover. The participant in the care recipient role confirmed via voice or touchscreen and retrieved the items, after which the robot returned to the charging station.

#### Beverage Delivery

This scenario comprised a standard and an extended sequence (phases 1 and 2). In phase 1, the participant in the nurse role configured deliveries for 2 rooms in the app using drop-down selection, loaded beverages into illuminated bottle holders at the loading area following robot guidance, and started execution on the touchscreen. The robot delivered to the room of the participant in the care recipient role using the standard arrival, person detection, confirmation, and pickup sequence. The second room delivery was conducted by study staff to complete a second standardized delivery sequence while keeping participant roles constant. In phase 2, 8 additional deliveries alternated between the 2 rooms. The participant in the care recipient role remained in one room and performed predefined interaction variations for deliveries to their room. These included a misunderstanding prompting repetition followed by rejection, as well as acceptance while using a walker and a wheelchair. In phase 2, the remaining deliveries were again handled by study staff. Rejected deliveries were documented in the app as not completed.

Across all scenarios, the participant in the nurse role performed typical documentation tasks in parallel to autonomous robot operation to reflect realistic time management practices. They also verified task completion in the app and documented the outcomes in accordance with the study protocol. Full scripts and step tables are provided in the supplementary material (Multimedia Appendix 1).

### Measures

With regard to sociodemographic variables, the following data were collected: age, gender, highest educational qualification, current occupation, and previous experience with robots.

In addition, the GAToRS was applied. The GAToRS is a validated multidimensional instrument that accounts for both positive and negative attitudes toward robots. It comprises four subscales: (1) personal level positive attitude (P+), (2) personal level negative attitude (P–), (3) societal level positive attitude (S+), and (4) societal level negative attitude (S–). Thus, the scale captures (1) perceived comfort and pleasure and (2) discomfort and fears toward robots on a personal level, along with (3) rational hopes and (4) rational fears on a societal level. The questionnaire shows Cronbach α coefficients ranging from 0.74 to 0.84 across the 4 scales [50].

Furthermore, the TUI was used. The TUI is a validated questionnaire used in technology acceptance research to capture evaluations and selected determinants of intention to use for emerging technologies, encompassing technology-related perceptions and user-related factors. Subscales were selected a priori in line with the study focus on the immediate postinteraction evaluation of the service robot. Accordingly, we administered the postuse subscales usability, usefulness, skepticism, and accessibility together with the separate ITU scale. Reported internal consistencies (Cronbach α) for these subscales range from 0.70 to 0.81 [51].

### Participants

A total of 30 participants took part in the study. The mean age was 43.2 (SD 11.2; range 22‐67) years. The sample consisted of 20 women and 10 men.

At the time of participation, 11 individuals were employed as geriatric nurses and 2 as geriatric nursing assistants. Additionally, 3 participants worked as nurses and another 3 as pediatric nurses. Six were employed as nursing assistants. Five participants had previously worked in nursing and were currently employed in health care delivery-related fields, including nursing education (n=1), case management (n=1), health care controlling (n=1), and research (n=2).

Almost one-third of participants (n=9) reported prior experience with robotics, either in private (n=7), professional (n=1), or other contexts (n=1).

### Data Analysis

Data were analyzed using IBM SPSS Statistics (version 29), applying both descriptive and inferential statistical methods. Within the GAToRS dataset, 10 missing items were identified without any discernible pattern. No variable exhibited more than 6.7% missing values. Given that previous studies have shown minimal differences in the performance of various imputation techniques when only 5%‐10% of values are missing [52,53], the use of simple imputation was deemed methodologically appropriate. Accordingly, mean imputation (series mean) was applied in SPSS.

Descriptive statistics are reported as means with SDs or, where appropriate, as medians with IQRs. As normal distribution could either not be assumed or was clearly absent, nonparametric tests were applied. Spearman rank correlation coefficient was used to assess statistical associations, and the Mann-Whitney *U* test was conducted for group comparisons. Statistical significance was set at *α*=.05, with correlations significant at the 0.01 level additionally highlighted.

### Ethical Considerations

The study was conducted in accordance with the Declaration of Helsinki and the International Council for Harmonisation Guideline for Good Clinical Practice [54]. The study protocol was reviewed and approved by the Ethics Committee at the Institute of Psychology, Humboldt-University, Berlin (2023‐14). All participants provided written informed consent prior to participation. Upon completion of the study, participants received financial compensation of EUR 30 (US $35). Privacy and confidentiality were ensured through data pseudonymization, secure storage of study data, and access restricted to authorized members of the research team.

## Results

### Participants’ General Attitude Toward Robots

**Figure 4.**
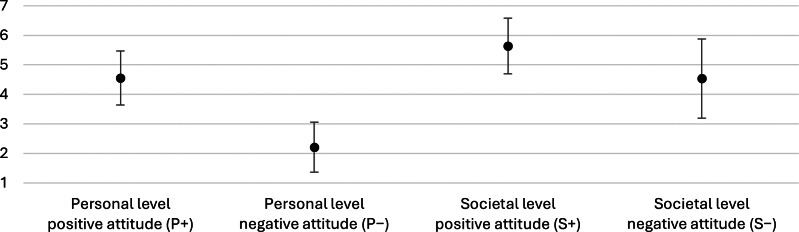
Results of the General Attitudes Towards Robots Scale subscales. The scale captures attitudes on two levels: personal (positive attitude=P+, negative attitude=P–) and societal (positive attitude=S+, negative attitude=S–). The x-axis displays the 4 subscales, and the y-axis shows the subscale scores. The dots represent the means, and the error bars indicate the SDs. The subscales range from 1 to 7. Higher values on P+ and S+ indicate stronger positive attitudes, whereas higher values on P– and S– indicate stronger negative attitudes.

To assess participants’ baseline attitudes toward robots, the GAToRS was applied (Figure 4). Each subscale yields scores from 1 to 7. Higher scores on P+ and S+ indicate stronger positive attitudes, whereas higher scores on P– and S– indicate stronger negative attitudes.

At the personal level, positive attitudes ranged from 2.6 to 6.6 (mean 4.6, SD 0.9), while negative attitudes ranged from 1 to 4 (mean 2.2, SD 0.8).

At the societal level, positive attitudes showed the highest mean value across all subscales, with scores ranging from 3.2 to 7 (mean 5.6, SD 0.9). In contrast, negative attitudes showed the greatest variability, with scores between 1.8 and 7 (mean 4.5, SD 1.3).

### Evaluation of the Developed Service Robot

**Figure 5.**
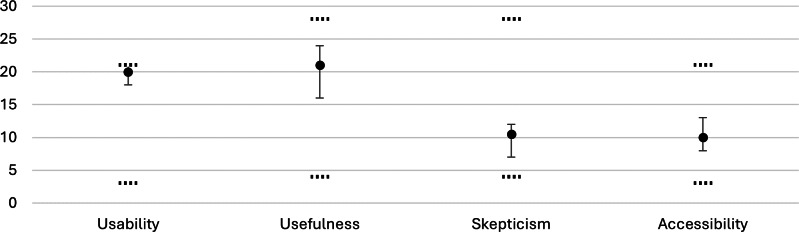
Results of the Technology Usage Inventory subscales. The x-axis displays the four subscales (usability, usefulness, skepticism, and accessibility), and the y-axis shows the subscale scores. The dots represent the medians, the error bars indicate the IQRs, and horizontal dotted lines mark the scale limits (usability and accessibility: 3‐21; usefulness and skepticism: 4‐28). Higher values indicate greater endorsement of the respective construct.

**Figure 6.**
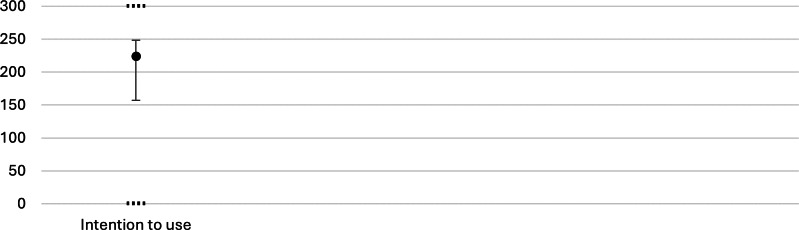
Results of the Technology Usage Inventory intention to use scale. The x-axis displays the scale, and the y-axis shows the score. The dot represents the median, the error bar indicates the IQR, and horizontal dotted lines mark the scale limits (0‐300). A higher value indicates a stronger intention to use the technology.

The general evaluation of the developed service robot was conducted using the TUI, focusing on the immediate postintervention responses. The results included the subscales usability, usefulness, skepticism, and accessibility (Figure 5), as well as the separate ITU scale (Figure 6). Higher scores indicate greater endorsement of the respective construct.

Usability scores were between 14 and 21, with a median of 20 (IQR 18-21). Usefulness showed the widest spread among the 4 subscales, with values from 6 to 28, a median of 21 (IQR 16-24). Scores for skepticism varied from 4 to 24, with a median of 10.5 (IQR 7-12). Accessibility was rated between 3 and 21, with a median of 10 (IQR 8-13). For the ITU scale, scores spanned from 0 to 300, with a median of 224.5 (IQR 157-248).

### Analysis of Dependencies and Influencing Factors

To measure the strength of the linear relationship between the participants’ general attitude toward robots, based on the GAToRS, and the dimensions of the TUI, a Spearman correlation analysis was computed (Table 1).

**Table 1.** Results of the Spearman correlation analysis between the General Attitudes Towards Robots Scale and the Technology Usage Inventory.

	Usability	Usefulness	Skepticism	Accessibility	Intention to use
Personal level positive attitude					
*r_s_*	0.057	0.549^a^	–0.317	0.457^b^	0.483^a^
*P* value	.76	.002	.09	.01	.007
N	30	30	30	30	30
Personal level negative attitude					
*r_s_*	–0.270	–0.359	0.436^b^	–0.309	–0.226
*P* value	.15	.052	.02	.10	.23
N	30	30	30	30	30
Societal level positive attitude					
*r_s_*	0.122	0.187	0.085	–0.072	0.179
*P* value	.52	.32	.66	.71	.34
N	30	30	30	30	30
Societal level negative attitude					
*r_s_*	–0.153	–0.387^b^	0.479^a^	–0.576^a^	–0.159
*P* value	.42	.03	.007	<.001	.40
N	30	30	30	30	30

a Correlation is significant at the 0.01 level (2-tailed).

b Correlation is significant at the 0.05 level (2-tailed).

P+ showed a significant positive correlation with perceived usefulness (*r_s_*(28)=0.549; *P*=.002), indicating a strong effect size [55]. Significant positive correlations were also found with accessibility (*r_s_*(28)=0.457; *P*=.01) and intention to use (*r_s_*(28)=0.483; *P*=.007), corresponding to medium effect sizes [55]. No significant correlations were observed with usability or skepticism.

P– correlated significantly positively with skepticism (*r_s_*(28)=0.436; *P*=.02), suggesting a medium effect size [55]. No significant correlations were found with usability, perceived usefulness, accessibility, or intention to use.

S+ did not exhibit any significant correlations with the dimensions of the TUI.

S– was significantly negatively correlated with perceived usefulness (*r_s_*(28)=−0.387; *P*=.03), indicating a medium effect size [55]. Additionally, a significant positive correlation was found with skepticism (*r_s_*(28)=0.479; *P*=.007), also reflecting a medium effect size [55]. A significant negative correlation was further observed with accessibility (*r_s_*(28)=−0.576; *P*<.001), corresponding to a strong effect size [55]. No significant correlations were found with usability or with intention to use.

To further examine associations between the TUI subscales (usability, usefulness, skepticism, and accessibility) and its ITU scale, an additional Spearman correlation analysis was conducted (Table 2).

**Table 2.** Results of the Spearman correlation analysis between the Technology Usage Inventory subscales (usability, usefulness, skepticism, and accessibility) and its intention to use scale.

	Intention to use
Usability	
*r_s_*	0.505^a^
*P* value	.004
N	30
Usefulness	
*r_s_*	0.740^a^
*P* value	<.001
N	30
Skepticism	
*r_s_*	−0.516^a^
*P* value	.004
N	30
Accessibility	
*r_s_*	0.628^a^
*P* value	<.001
N	30

a Correlation is significant at the 0.01 level (2-tailed).

Usability (*r_s_*(28)=0.505; *P*=.004), perceived usefulness (*r_s_*(28)=0.740; *P*<.001) and accessibility (*r_s_*(28)=0.628; *P*<.001) showed significant positive correlations with intention to use. Skepticism, however, yielded a significant negative correlation (*r_s_*(28)=−0.516; *P*=.004). All 4 correlation coefficients exceeded the threshold of 0.5, indicating strong effect sizes according to Cohen's criteria [55].

Table 3 presents the comparison of the TUI subscale scores between participants with and without prior robotics experience, analyzed using the Mann-Whitney *U* test.

**Table 3.** Results of the Technology Usage Inventory by robotics experience.

TUI^a^ subscales and robotics experience	Participants, n	Median (IQR)	*U* test	*z*-score	*P* value
Usability					
No	21	20 (18-21)	73	−1.019	.31
Yes	9	21 (19-21)			
Usefulness					
No	21	22 (16-23)	82	−0.567	.57
Yes	9	21 (18-26)			
Skepticism					
No	21	11 (7-13)	75.5	−0.865	.39
Yes	9	8 (7-11)			
Accessibility					
No	21	11 (8-13)	93.5	−0.045	.96
Yes	9	9 (8-12)			
Intention to use					
No	21	211 (157-247)	83.5	−0.498	.62
Yes	9	241 (166-269)			

a TUI: Technology Usage Inventory.

Usability was rated slightly higher by participants with prior experience in robotics (median 21, IQR 19-21) compared to those without (median 20, IQR 18-21). Yet, this difference did not reach statistical significance (*U*=73; *P*=.31).

Perceived usefulness was also rated comparably across both groups, with slightly higher scores among participants without prior robotics experience (median 22, IQR 16-23) than among those with experience (median 21, IQR 18-26), but this difference also failed to achieve statistical significance (*U*=82; *P*=.57).

Regarding skepticism, participants with prior robotics experience reported lower levels (median 8, IQR 7-11) compared to those without (median 11, IQR 7-13). Similarly, this difference was not statistically significant (*U*=75.5; *P*=.39).

Accessibility was perceived as slightly higher by participants without prior robotics experience (median 11, IQR 8-13) relative to those with experience (median 9, IQR 8-12). Once again, this difference did not attain statistical significance (*U*=93.5; *P*=.96).

Finally, the intention to use was higher among participants with prior robotics experience (median 241, IQR 166-269) than among those without (median 211, IQR 157-247). However, the difference on this scale was not statistically significant either (*U*=83.5; *P*=.62).

## Discussion

### Summary of Key Findings

This study investigated nursing staff’s general attitudes toward robotics, as well as their evaluation of and intention to use a newly developed service robot intended to support routine tasks in inpatient care settings. The quantitative analyses revealed a differentiated pattern of findings.

The GAToRS was used to assess participants’ perceptions of robots. Ratings on the P+ subscale, which measures trust in robot developers, trust in robots, and comfort in interacting with robots, were moderately high across the sample. Consistent with this, ratings on the P– subscale, assessing discomfort with using robots, apprehension about misunderstandings, and unease in physical proximity to robots, were low and showed little variability. Responses on the S+ subscale, which captures perceived societal benefits of robots such as task delegation, safety, and assistance in meaningful activities, were consistently high. The S– subscale, which includes concerns about job displacement, reduced human interaction, and the necessity for regulatory oversight, yielded moderate ratings with a broader distribution of responses compared to S+.

The evaluation of the developed service robot was carried out using the TUI, focusing specifically on the subscales usability, perceived usefulness, skepticism, accessibility, and the ITU scale. Participants reported high ratings on usability, which measures the extent to which the robot is perceived as user-friendly and easy to operate. Similarly, ratings on the usefulness subscale, reflecting the perceived practical benefit of the robot in supporting everyday activities, were consistently high across the sample. The skepticism subscale, which captures distrust, perceived risks, and critical attitudes toward the technology, received low ratings. In contrast, accessibility, which assesses perceived affordability and the ease with which the robot can be acquired, was rated lower compared to the other dimensions. Participants’ ratings on the ITU scale, measuring the reported willingness to acquire and use the robot, were high.

Correlation analyses integrating the GAToRS demonstrated that individually positive attitudes toward robots were associated with higher perceived usefulness and accessibility, as well as a stronger intention to use the system. Individually negative attitudes were primarily linked to elevated skepticism. On the societal level, negative perceptions were associated with increased skepticism and diminished ratings of usefulness and accessibility. Notably, perceived societal optimism toward robots showed no statistically significant associations with any of the TUI dimensions.

Further correlational analyses identified multiple statistically significant linear relationships between dimensions of the TUI and the behavioral intention to use the service robot. Usability, usefulness, and accessibility were positively associated with intention to use, whereas skepticism correlated negatively.

In addition, prior experience with robotics did not exert a significant influence on either the evaluation of the developed system or the intention to use it.

### Interpretation of Key Findings

The positive evaluation and high intention to use the developed service robot may be attributable to the user-centered development approach adopted. Within the framework of the RoMi project, the objective of designing the system through the active participation of nursing staff was successfully achieved. The robot was iteratively refined, both technically and with regard to its application scenarios, based on insights from prior empirical studies [19,46-49]. The early and continuous integration of end users facilitated a development trajectory that was aligned with the actual demands of everyday nursing practice, in contrast to predominantly technology-driven design strategies. The favorable assessments of usability and usefulness, as well as the pronounced intention to use the system, thus provide empirically grounded support for the effectiveness of participatory design approaches, consistent with findings from previous research [5,19,41,46].

Analyses of the TUI dimensions further demonstrate that usability, usefulness, accessibility, and skepticism all serve as strong predictors of the intention to use. These findings extend beyond the core constructs of the TAM and underscore the necessity of adapting and expanding the model for health care contexts through the inclusion of additional context-sensitive factors [29].

The strong positive correlation identified between usability and the intention to use the developed robot aligns with established findings in technology acceptance research. Usability has consistently been shown to be a critical determinant of adoption in health care contexts, particularly among nursing staff operating under time constraints [56]. Technologies that are intuitive and easy to integrate into nursing workflows are more likely to be accepted and used consistently [5,57].

Similarly, the strong positive correlation between perceived usefulness and intention to use reflects the importance of demonstrable benefits in practice. In nursing, adoption decisions are often contingent on clear advantages to patient care and workflow efficiency [28]. Robots that reduce workload and improve care outcomes are more likely to gain acceptance [21]. These findings indicate that pilot implementations and use-case demonstrations play a key role in fostering both perceived usefulness and adoption in nursing environments.

The strong positive correlation between perceived accessibility and intention to use further highlights the relevance of economic and logistical considerations in adoption decisions. The data suggest that nursing staff are more likely to engage with robotic systems when these are perceived as affordable and readily available. This finding is consistent with previous research identifying cost-related factors, such as initial investment and maintenance expenses, as potential barriers to implementation [21,58]. Strategies that improve economic accessibility, including subsidies, leasing models, or evidence of long-term cost-effectiveness, may therefore be critical to facilitate broader integration into nursing practice.

The strong negative correlation identified between skepticism and the intention to use the developed service robot underscores the importance of psychological and attitudinal barriers in shaping acceptance. In this study, skepticism was operationalized through items addressing perceived risks, potential disruption to daily routines, and a negative balance of expected benefits and drawbacks. These findings are consistent with previous research showing that concerns related to safety, reliability, and professional roles can hinder the acceptance of robotic systems [22]. Addressing such concerns requires more than technical refinement: regulatory clarity, comprehensive training, and transparent communication about system capabilities and limitations are considered essential [21,22,59], with particular emphasis on framing robotic systems as supportive rather than substitutive.

The findings related to the GAToRS clearly differentiate between societal and individual levels. The subscale S+ reflects an abstract acknowledgment of the potential societal benefits of assistive robotic systems within the surveyed sample. However, S+ is not significantly associated with the evaluation of the developed service robot or with the individual intention to use it. This dissociation may suggest that an intellectual consensus on the societal benefits of robotics is not sufficient to translate into concrete behavioral readiness within the everyday practice of nursing.

In contrast, S– exhibits a substantially stronger association with the evaluation of the service robot. The subscale is significantly negatively correlated with the perceived usefulness and accessibility of the system and positively correlated with skepticism. Similar to S+, S– does not exert a direct influence on the individual intention to use. However, this asymmetry between S+ and S– may reflect a negativity bias, whereby societal-level concerns impair specific perceptions of the robot, while positive societal-level attitudes remain largely ineffective in shaping individual evaluations [60]. Accordingly, implementation strategies should not only emphasize potential benefits but also proactively address negative societal narratives—for example, through transparent communication that highlights the robot’s role in supporting nursing staff, as well as clear information on potential risks and the corresponding safety measures [59].

At the individual level, a contrasting pattern emerges. P+ is positively associated with perceived usefulness and accessibility of the service robot, and also correlates with a stronger intention to use. As expected, nursing staff who reported a generally favorable disposition toward robotics evaluated the developed service robot more positively and demonstrated a higher intention to use it in future practice.

Although scores on P– were generally low in this sample, the subscale showed a significant positive correlation with skepticism. However, no significant associations were observed with usability, perceived usefulness, accessibility, or intention to use. Once again, an asymmetry becomes apparent. While positive dispositions are associated with more favorable evaluations across multiple outcome variables, negative dispositions are primarily linked to increased skepticism. Notably, this asymmetry contrasts with the pattern observed at the societal level, where negative attitudes exert a broader influence. In this sense, the expected negativity bias appears attenuated at the individual level, suggesting that favorable personal attitudes toward robotics may be more impactful in shaping evaluative outcomes than negative ones.

In summary, the data suggest that societal-level attitudes (S+ or S–) may shape the broader cognitive context in which robotic systems are perceived, whereas individual-level factors (P+ or P–) and concrete user experiences are ultimately more decisive for actual readiness to adopt such technologies. From a practical perspective, these findings underscore the importance of proactively counteracting negative societal narratives surrounding robotics—especially by addressing concerns about job displacement and by ensuring transparent communication of potential risks and existing safety measures, particularly those concerning care recipients [21,41]. In addition, positive individual experiences with robotic systems should be facilitated at an early stage, for example, through hands-on sessions or pilot implementations at the ward level [5,41]. A combined approach that integrates risk-sensitive communication with experience-based benefit framing constitutes a promising strategy for fostering the acceptance of service robots in nursing practice.

Another noteworthy observation is the unexpectedly limited influence of prior experience with robotics on the intention to use the developed service robot. In contrast to previous studies that describe prior exposure as a facilitator of technology acceptance [31,38,44], the present results show no statistically significant differences in the evaluation of usability, usefulness, skepticism, accessibility, or intention to use. Although minor trends in the expected direction were observable, the absence of significant group differences may be attributable to several explanatory factors, including structural characteristics of the research and development project and aspects of its methodological implementation. First, the user-centered and participatory development process likely enhanced the system’s approachability across experience levels. The continuous involvement of nursing staff throughout the design and refinement phases may have resulted in a solution that is intuitively operable, useful, and closely aligned with everyday nursing practice—even for users without prior exposure. Second, the selected application scenarios should have addressed tasks that are broadly accepted and perceived as meaningful within the professional nursing context. When the service robot’s intended use is seen as relevant and beneficial, prior technical familiarity may become less decisive for acceptance. Third, the standardized, hands-on training provided to all participants ensured a uniform introduction to the system and likely helped reduce disparities in user confidence and system understanding. As a controlled intervention, this training could have mitigated differences in prior experience, thereby contributing to a more consistent evaluation of the system across groups.

These considerations indicate that a lack of prior experience does not necessarily constitute a major barrier to acceptance, especially when supportive conditions are present. Such conditions may involve user-centered system and scenario design that enables experience-independent access, along with targeted training efforts to reduce uncertainty and strengthen user competence.

### Limitations

The interpretations of these findings are informed by methodological considerations. The sample size of 30 participants was adequate for an exploratory study and allowed for the identification of relevant patterns. However, it inherently limits the generalizability of the results. Subtle effects, such as potential associations with prior robotic experience, may not have been captured.

Additionally, a potential selection bias cannot be ruled out. Given that participation was voluntary, individuals with a greater affinity for technology may have been overrepresented, which could have contributed to a more favorable evaluation of the developed service robot. While such bias cannot be entirely avoided in applied research, it should be considered when interpreting the observed positive ratings.

Furthermore, the controlled laboratory setting, while beneficial for standardization and internal validity, only partially captures the complexity and situational variability of everyday nursing practice. Similarly, the relatively brief interaction period with the service robot prevents conclusions about sustained use or long-term integration into professional routines.

While acknowledging the study’s limitations, this paper lays a foundation for future research by demonstrating the system’s readiness for field testing and offering valuable insights into the factors influencing nursing staff’s acceptance of service robots.

### Conclusion

This study investigated a service robot developed to support routine tasks in inpatient care. A total of 30 nurses tested the system in 3 application scenarios (information service, item delivery, and beverage delivery), alternating between the roles of nurse and care recipient. Overall, the robot was evaluated positively, with high ratings for usability and perceived usefulness, low levels of skepticism, and moderate accessibility. The reported intention to use the system was high, indicating a substantial level of acceptance and a likely readiness for practical use in the tested application scenarios. The findings thus confirm the relevance of both the selected scenarios and the robot’s potential to support these tasks.

The significant correlations observed between acceptance-related dimensions and the intention to use underline the necessity of a multilayered strategy that extends beyond technical optimization. User-centered design processes, transparent communication regarding system capabilities and limitations, as well as opportunities for personal orientation and hands-on experience, are essential to align robotic systems with the practical requirements, expectations, and concerns of nursing staff.

As the field of robotics in nursing advances, these aspects should be systematically integrated into future research, development, and nursing education. Ultimately, such an approach is essential to ensuring not only that the deployment of service robots in nursing is technically feasible but also that their potential to alleviate the physical and cognitive burden on nursing staff is fully realized, thereby enabling their effective and sustainable integration into the future of nursing practice.

## Supplementary material

10.2196/86824 Multimedia Appendix 1 Application scenarios.

## Abbreviations

GAToRS: General Attitudes Towards Robots Scale
ITU: intention to use
P+: personal-level positive attitudes
P–: personal-level negative attitudes
RoMi: *Roboterunterstützung bei Routineaufgaben zur Stärkung des Miteinanders in Pflegeeinrichtungen* (translation: robotic support for routine tasks to strengthen collaboration in care facilities)
S+: societal-level positive attitudes
S–: societal-level negative attitudes
TAM: technology acceptance model
TUI: Technology Usage Inventory
