# Supplementary material for: Nurses’ Evaluation of a Service Robot for Inpatient Care: Technology Acceptance Study
Source: JMIR Nurs. 2026 Apr 14;9:e86824. doi: 10.2196/86824 (PMC13078706; doi:10.2196/86824)
Supplement: Multimedia Appendix 1 [file nursing-v9-e86824-s001.pdf]

SCENARIO 1 – INFORMATION SERVICE:

In this scenario, the robot was used to deliver a notification to a care recipient. Participants assigned to the nurse role were instructed to set up a notification using a smartphone application. This involved selecting the designated patient room within the app and entering the information into a free-text field.

Once the task was submitted, the robot autonomously navigated to the assigned patient room. Upon arrival, it signaled its presence by emitting a bell sound and waited until a person entered its detection area. At this point, the participant in the care recipient role was asked to approach the robot at the door and confirm receipt of the message.

Once a person was detected, the robot initiated an interaction by greeting the participant and asking if they wished to receive a notification. Upon confirmation, either via voice input or touchscreen interaction, the robot delivered the message both audibly and visually on its integrated touchscreen display. An option to repeat the notification was also provided. If no repetition was requested, the robot autonomously returned to its charging station.

To complete the scenario, the participant in the nurse role was required to verify task completion using the smartphone application and to document the result in accordance with the study protocol.

Step	Nurse (Participant A)	Robot	Care recipient (Participant B)
1	Opens smartphone application	–	–
2	Selects patient room and enters notification text	–	–
3	Sends task to robot	Waits for task	–
4	–	Navigates to assigned patient room	–
5	–	Signals arrival (bell sound)	Waits for cue
6	–	Waits for detection of person	Approaches robot (enters detection zone)
7	–	Detects person and initiates interaction	Listens
8	–	Greets and asks for confirmation	Confirms via voice or touchscreen

9	–	Delivers notification (voice and display)	Receives message
10	–	Offers message repetition	Optionally requests message repetition
11	–	Returns to charging station	–
12	Verifies task completion in app	–	–
13	Documents task status	–	–

SCENARIO 2 – ITEM DELIVERY:

In this scenario, the participant in the nurse role was tasked with initiating a delivery using the smartphone application. To do so, the designated patient room was selected within the app, and the relevant items to be delivered were entered into a free-text field.

Upon task submission, the robot autonomously navigated to a predefined loading area. Once it reached the designated position, it waited until a person entered its detection area to initiate the loading interaction. When the participant in the nurse role approached, the robot started the loading sequence by providing instructions via voice output and its touchscreen display. The participant then placed the physical items on the tray located on the rear side of the robot. After confirming that the loading was complete by pressing a start button on the touchscreen, the robot proceeded to autonomously navigate to the assigned patient room.

Upon arrival, it signaled its presence by emitting a bell sound and waited until a person entered its detection area. The participant in the care recipient role was instructed to approach the robot and engage in the delivery interaction. Once detected, the robot greeted the participant and asked whether they wished to receive the items. Confirmation could be given either via voice input or by interacting with the touchscreen interface. Following confirmation, the robot instructed the participant to retrieve the items from its tray.

After the handover was performed and acknowledged, the robot autonomously returned to its charging station.

To complete the scenario, the participant in the nurse role was required to verify task completion using the smartphone application and to document the result in accordance with the study protocol.

Step	Nurse (Participant A)	Robot	Care recipient (Participant B)
1	Opens smartphone application	–	–
2	Selects patient room and enters item list	–	–
3	Sends delivery task to robot	Receives task and starts navigation	–
4	–	Navigates to loading area	–
5	–	Waits for person in detection area	–
6	Enters detection zone	Starts loading interaction (voice and touchscreen display)	–
7	Places items on tray	–	–
8	Starts task execution via touchscreen display	Starts navigation to patient room	–
9	–	Signals arrival (bell sound)	Waits for cue
10	–	Waits for person in detection area	Approaches robot
11	–	Detects person and initiates interaction	Listens
12	–	Greets and asks for confirmation	Confirms via voice or touchscreen
13	–	Prompts item pickup	Retrieves items and confirms receipt
14	–	Returns to charging station	–
15	Verifies task completion in app	–	–
16	Documents task status	–	–

SCENARIO 3 – BEVERAGE DELIVERY:

This scenario involved the delivery of beverages to multiple patient rooms using the robot.

Phase 1 – Standard Beverage Delivery:

The first task required the robot to deliver beverages to two patient rooms. To initiate the task, the participant in the nurse role selected the designated patient rooms in the smartphone application and specified the desired drink for each recipient using a drop-down menu. After confirming the order, the robot autonomously navigated to the designated loading area.

Upon arrival, it waited for a person to enter its detection zone in order to start the loading interaction. Once the participant entered this zone, the robot provided step-by-step loading instructions via voice

and touchscreen display. After placing the beverages in the required illuminated bottle holders, the participant initiated the delivery by confirming on the touchscreen display.

The robot then navigated to the first patient room, signaled its presence with a bell sound, and waited until someone entered its detection area. At this point, the participant in the care recipient role was asked to approach the robot and interact with it to receive the beverage. Upon detection, the robot greeted the participant and asked whether they wished to receive the assigned drink. Confirmation could be given either via voice or touchscreen display. The robot then prompted the person to remove the beverage from the required illuminated bottle holder.

After this interaction, the robot proceeded to the second patient room, where the procedure was repeated by a member of the study staff.

Following both deliveries, the robot returned to its charging station.

To complete the scenario, the participant in the nurse role was required to verify task completion using the smartphone application and to document the result in accordance with the study protocol.

Phase 2 – Extended Beverage Delivery:

In the extended version of the scenario, the task was expanded to comprise eight beverage deliveries and additional variations of human-robot interaction on the part of the participant in the care recipient role. Since only two patient rooms were available, deliveries alternated between these same rooms. The participant in the nurse role was again responsible for configuring the deliveries in the application and loading the robot accordingly. The participant in the care recipient role remained in the same room throughout the sequence. The variations in human-robot interaction during this phase were as follows:

- First delivery: Acceptance via one of the available input channels (voice or touchscreen display).
- Third delivery: The participant was instructed to simulate a misunderstanding, prompting the robot to repeat its delivery offer, which was then to be rejected.
- Fifth and seventh deliveries: The beverage was to be received using a walker and a wheelchair, respectively, to simulate assisted mobility interactions.
- Remaining deliveries: Interactions were carried out by study staff

Upon completion of the final delivery, the participant in the nurse role verified task completion using the smartphone application and documented the outcome in accordance with the study protocol (four deliveries were marked as not completed, with comments indicating that the care recipients had declined to accept the beverage).

Phase 1:

Step	Nurse (Participant A)	Robot	Care recipient (Participant B)
1	Opens smartphone application	–	–
2	Selects 2 patient rooms and drink types	–	–
3	Confirms beverage delivery task	Receives task and starts navigation	–
4	–	Navigates to loading area	–
5	–	Waits for person in detection area	–
6	Enters detection zone	Starts loading interaction (voice and screen)	–
7	Places beverages in the required illuminated bottle holders	Provides loading instructions and illuminates the required bottle holders	–
8	Starts task execution via touchscreen	Navigates to first patient room	–
9	–	Signals arrival (bell sound)	Waits for cue
10	–	Waits for person in detection area	Approaches robot
11	–	Detects person and initiates interaction	Listens
12	–	Greets and asks if a beverage is desired	Confirms via voice or touchscreen
13	–	Prompts beverage pickup	Retrieves beverage from required illuminated bottle holder
14	–	Navigates to second patient room	–
15	–	Repeats delivery and interaction process (handled by study staff)	–
16	Verifies task completion in app	–	–
17	Documents task status	–	–

Phase 2:

Step	Nurse (Participant A)	Robot	Care recipient (Participant B)
1	Opens smartphone application	–	–
2	Selects 8 patient rooms and drink types	–	–
3	Confirms beverage delivery task	Receives task and starts navigation	–
4	–	Navigates to loading area	–
5	–	Waits for person in detection area	–
6	Enters detection zone	Starts loading interaction (voice and screen)	–
7	Places beverages in the required illuminated bottle holders	Provides loading instructions and illuminates the required bottle holders	–
8	Starts task execution via touchscreen	Navigates to first patient room	–
9	–	Signals arrival (bell sound)	Waits for cue
10	–	Waits for person in detection area	Approaches robot
11	–	Detects person and initiates interaction	Listens
12	–	Greets and asks if a beverage is desired	Confirms via voice or touchscreen
13	–	Prompts beverage pickup	Retrieves beverage from required illuminated bottle holder
14	–	Navigates to second patient room	–
15	–	Repeats delivery and interaction process (handled by study staff)	–
16	–	Returns to first patient room and initiates delivery 3	Simulates misunderstanding
17	–	Repeats offer	Rejects delivery
18	–	Navigates to second patient room and initiates delivery 4 (handled by study staff)	–
19	–	Returns to first patient room (delivery 5)	Approaches with walker, accepts drink
20	–	Navigates to second patient room and initiates delivery 6 (handled by study staff)	–
21	–	Returns to first patient room (delivery 7)	Approaches with wheelchair, accepts drink

22	–	Navigates to second patient room and initiates delivery 8 (handled by study staff)	–
23	Verifies task completion in app	–	–
24	Documents task status	–	–

Across all three scenarios, participants assigned to the nurse role were instructed to perform typical, exemplary nursing documentation tasks in parallel while the robot was executing its assigned actions. This was intended to reflect realistic time management practices in everyday nursing settings, where available time would be used efficiently during autonomous robot operations.
